# Strategies for updating rules driven by reinforcement learning to solve social dilemmas

**DOI:** 10.1371/journal.pone.0341925

**Published:** 2026-03-10

**Authors:** Yang Wang, Xingchen Yu, Shounan Lu

**Affiliations:** 1 School of Information and Control Engineering, North China Institute of Science and Technology, Langfang, Hebei, China; 2 Key Laboratory of Brain-Computer Interface Technology Application of the Ministry of Emergency Management, Langfang, Hebei, China; 3 School of Mathematical Sciences, Laboratory of Mathematics and Complex Systems, MOE, Beijing Normal University, Beijing, China; University of Electronic Science and Technology of China, CHINA

## Abstract

This study incorporates historical performance into traditional imitation rules and proposes a moderated strategy update rule. In this framework, an individual’s temporal historical performance is calculated using the BM model. By adjusting the parameter *δ*, the influence of historical performance on strategy learning is determined, and the evolution of cooperation is subsequently observed. Results show that the proposed strategy update rule promotes cooperation more effectively than the traditional version, and systemic cooperation is further enhanced as *δ* increases. The reason why the proposed rule enhances cooperation is that it amplifies the evaluation of cooperative behavior while compressing the evaluation of defective behavior. Although establishing system objectives may hinder the diffusion of cooperative behavior, appropriate performance evaluation mechanisms can mitigate this adverse effect. Our results indicate that multidimensional evaluation can provide a theoretical basis for explaining cooperative behavior in complex environments.

## 1. Introduction

Cooperation is fundamental to the stability of biological ecosystems and human social systems. Yet, reconciling cooperative behaviors with Darwinian principles of natural selection [1] presents a persistent theoretical challenge. This has prompted most researchers to explore the principles underlying cooperation. Nowak’s seminal work summarizes five key mechanisms facilitating cooperation: kin selection, direct reciprocity, indirect reciprocity, network reciprocity, and group selection [2]. Crucially, network reciprocity exploits underlying population structure, enabling cooperators to cluster and thereby sustain cooperation [3]. Subsequent research has extended this foundational framework, incorporating diverse mechanisms such as punishment and reward [4–7], environmental feedback [8,9], social diversity [10], teaching activities [11,12], reputation [13], among others [14].

While numerous studies posit strategy learning in cooperative evolution primarily through imitation rules [3]—where benefits serve as the key driver—the reality involves complex, multidimensional influences. Strategy learning behavior results from the interplay of factors like self-learning and social learning [15]. Scholars have explored the impact of diverse strategy updating rules on the emergence and maintenance of cooperation. For instance, Yan and Hui demonstrated that integrating reputation mechanisms significantly enhances cooperation [16]. Similar conclusions were reached by Zhang et al. and He et al. [17,18]. Lu and Wang, incorporating past-performance into learning rules, found that increasing the weight of historical outcomes progressively strengthens system-wide cooperation [19]. Other investigated rules include popularity-driven [20] and experience-driven updates [21]. Collectively, these findings indicate that multi-factor dependent learning rules generally foster cooperative evolution.

Despite this extensive exploration, the predominant focus remains on extrinsic social attributes, such as reputation. Consequently, a critical gap persists: the evolutionary patterns and outcomes of system cooperation under composite strategy learning rules driven primarily by intrinsic individual attributes remain unexplored.

In addition, most existing studies focus on the immediate benefits of individual behavior within a single interaction round, overlooking the accumulated experience from prior games. This approach fails to capture the natural phenomenon in which organisms adapt their social strategies based on environmental cues, including feedback from past experiences. Reinforcement learning (RL) rules, however, effectively incorporate the cumulative influence of such memory effects [22–24]. Consequently, researchers have increasingly explored RL in evolutionary cooperation studies. For instance, Jia et al. demonstrated that incorporating RL enhances system-wide cooperation [25]. However, the research conducted by Lu et al [19], focused on the impact of reinforcement learning based relationship strength adjustment on cooperation, neglecting the dual effects of internal and external factors in strategy learning. The studies of Jia et al. [25] and Geng et al. [26] also overlooked the role of individual intrinsic factors in strategy learning. In addition, although Zhang et al. combined reinforcement learning with consensus learning rules to study how different policy update mechanisms affect cooperative evolution, the model assumes that individuals are rational [27,28], which does not reflect the actual cooperative evolution situation. Notably, recent findings suggest RL not only accounts for conditional cooperation but also explains patterns of emotional reciprocity [29,30].

Accordingly, we conceptualize the system’s consistency goal [31–35] as an intrinsic driver of individual behavior. Achieving this goal serves as one criterion for evaluating behavioral performance: success in the preceding round raises the current performance score, while failure lowers it. Drawing on reinforcement learning principles, we accumulate behavioral information across successive rounds to assess historical performance. From a global perspective, we evaluate individual performance and use the resulting assessment as a measure of social evaluation. By taking the interactive payoffs among individuals as the basis for mutual assessment, the strategy learning process is systematically guided through the integration of both social and individual evaluations. On this basis, we examine the evolution of cooperation within the system. This update rule incorporates both real-time game payoffs and historical behavior to govern strategy revisions. Our results show that this modified update mechanism significantly promotes the emergence of prosocial behaviors in the system.

## 2. Model

In this work, the weak Prisoner’s Dilemma is used [3], and without loss of generality, set the game payoff *T* = *b* (*b* ＞ 1), *R* = 1, and *P* = *S*=0, and follows: *T* > *R* > *P* > *S* and 2*R* > *T* + *S*. The corresponding payoff matrix *M* in Eq. (1).


$$\begin{array}{c} M=\left(\begin{array}{cc} @cc@R=1 & S=0\\ T=b & P=0 \end{array}\right) \end{array}$$ (1)

Then, we construct a two-dimensional spatial network with periodic boundary to depict the relationships between individuals in the system. Initially, each individual randomly adopts either cooperation (*S*_*i*_ = *C*) or defection (*S*_*i*_ = *D*) with equal probability *p*, as specified in Eq. (2), interacting with its four nearest neighbors, accumulating income *P*_*i*_ based on Eq. (3), where *Ω*_*i*_ is the set of individual *i’s* neighbors.


$$S_{i}=C=(1,0{)}^{T},S_{j}=D=(0,1{)}^{T}$$ (2)

$$P_{i}=\sum_{j\in \Omega i}S_{i}^{T}MS_{j}$$ (3)

Subsequently, the BM model [22–24] is employed within a reinforcement learning framework to calculate and assess an individual’s historical performance. In performance evaluation, the system’s aspiration level for consistency serves as the evaluation benchmark. If an individual’s cumulative performance during the evaluation period reaches or exceeds this benchmark, their score increases; otherwise, it decreases. And this adjustment mechanism operates persistently. Within BM reinforcement learning, the performance evaluation process occurs in two distinct steps. First, performance is scored according to the degree of deviation between the cumulative revenue and the expected system consistency target, in Eq. (4), where *β* (*β*  ≥ 0) is the stimulus sensitivity to the reinforcement signal of (*P*_*i*_-A). Subsequently, evaluating based on individual strategies, in Eq. (5), where *g*_*i*_ represents the satisfaction of players with the difference between *P*_*i*_ and A, then, the global evaluation results *E*_*i*_ of individual historical behavior performance can be quantified, with the calculation formula specified as follows.


$$g_{i}=\tanh[\beta (P_{i}-A)]$$ (4)

$$E_{i}(t+1)=\left\{ \begin{array}{c} @l@E_{i}(t)+[1-E_{i}(t)timesg_{i},\;if\;S_{i}(t)=C\text{\textbackslash{} \textbackslash{} }and\;g_{i}\geq 0\\ E_{i}(t)+E_{i}(t)\times g_{i},\;if\;S_{i}(t)=C\text{\textbackslash{} \textbackslash{} \textbackslash{} \textbackslash{} \textbackslash{} \textbackslash{} \textbackslash{} \textbackslash{} \textbackslash{} \textbackslash{},\textbackslash{},}and\;g_{i}<0\\ E_{i}(t)-E_{i}(t)\times g_{i},\;if\;S_{i}(t)=D\text{\textbackslash{} \textbackslash{} \textbackslash{} \textbackslash{} \textbackslash{} \textbackslash{} \textbackslash{} \textbackslash{} \textbackslash{} \textbackslash{} \textbackslash{},}and\;g_{i}\geq 0\\ E_{i}(t)-[1-E_{i}(t)]\times g_{i},\;if\;S_{i}(t)=D\text{\textbackslash{} \textbackslash{} \textbackslash{} \textbackslash{},}and\;g_{i}<0 \end{array}\right.$$ (5)

Where, parameter *A* represents the consistency goal or expected level of the system, and define *A* = *k*_*i*_*α*, where *k*_*i*_ = 4 denotes player *i*’s degree, representing the number of their four nearest neighbors [36,37], and *α* signifies the system’s consistency aspiration or goal level.

Finally, to refine learning strategies, this study proposes a moderated update rule. This rule integrates individual historical performance assessment with imitation dynamics, combining game payoff and historical performance to guide strategy updates, refers to the previous strategy learning rule setting and linearly adds them together [38,39]. The parameter *δ* (*δ* ∈ [0, 1]) modulates the weighting of historical performance in learning. When *δ* = 0, the strategy learn rule returns to its traditional version [3]. When *δ* > 0, both game payoffs and historical performance jointly shape strategy adaptation dynamics. Specifically, during the strategy update or learning process, the focal individual *i* will randomly select a nearest neighbor *j* and decide whether to learn the strategy of neighbor *j* based on probability *W* that based on Eq. (6). Here, parameter *K* quantifies the stochastic noise level that enables irrational decisions. As *K* → 0, agent *i* deterministically adopts the strategy of adjacent agent *j*, whereas when *K*→∞, the strategy imitation occurs randomly. Following Ref. [40], we set *K* = 0.5.


$$W_{\left(S_{i}(t)\leftarrow S_{j}(t)\right)}=\frac{\text{1}}{1+\exp[-\frac{\left(P_{i}-P_{j})+\delta *(E_{i}(t)-E_{j}(t)\right)}{K}]}$$ (6)

To evaluate the effectiveness of the proposed cooperation-enhancing mechanism, Monte Carlo simulations (*MCS*, which stands for Monte Carlo step) were performed on a 200 × 200 lattice network. Initially setting *p* = 0.5 and *E*_*i*_ (0) =0.5, each individual updated their strategy once per full *MCS* cycle on average. The equilibrium cooperation frequency *fc* was measured at 1 × 10^4^ *MCS*, with data averaged over 3 × 10^3^ *MCS* to minimize fluctuations. Results reflect 20 independent trials.

## 3. Result

**Fig 1:**
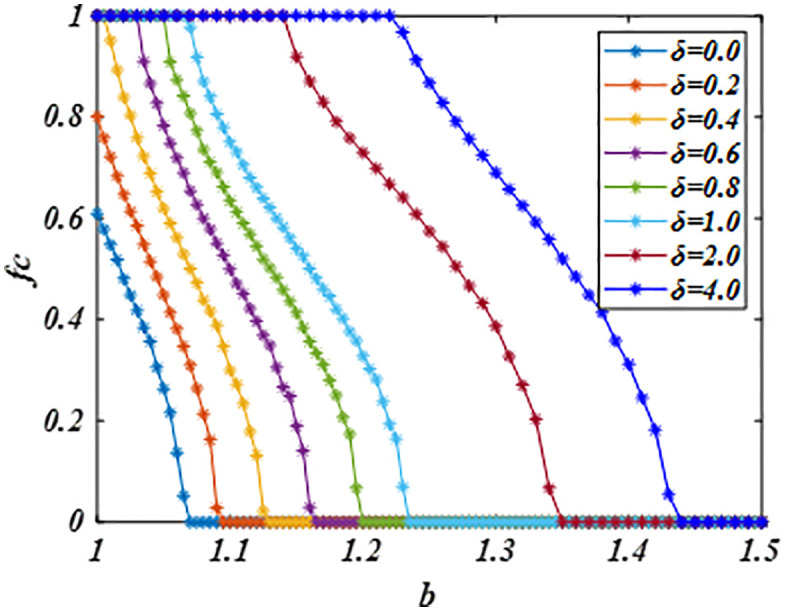
Illustrates *fc* versus *b* under different *δ* values. Vertically, with increasing *δ*, the system evolves to a stable state with a greater degree of cooperation. Showing that the proposed mechanism promotes cooperation and broadens its disappearance threshold *b*. Parameters are set to *β* = 2, *α* = 0.5 based on Ref. [24] and *L* = 200.

Fig 1 illustrates the evolution of cooperation level *fc* across defect temptation values *b* for varying *δ*. When *δ* = 0, strategy updates revert to conventional imitation rules, causing cooperators to rapidly disappear even at low *b*. For *δ* > 0, however, agents incorporate historical performance into strategy evaluation. This modification substantially elevates cooperation levels, with higher *δ* values further amplifying cooperative behavior. In particular, when *δ* is set to 4, the payoff dimensions become consistent with historical performance, promoting a greater degree of system cooperation. Consequently, the moderated update rule extends the critical threshold *b* for cooperation extinction beyond conventional imitation, thereby promoting both the emergence and sustainability of cooperation.

**Fig 2:**
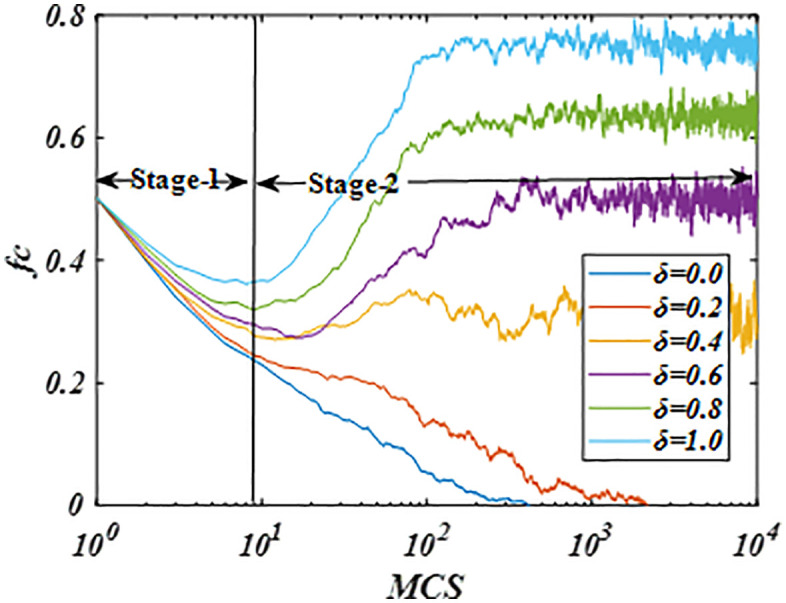
Illustrates temporal evolution of cooperative for some *δ* values, showing that the proposed mechanism can promote cooperation. With increasing *δ*, the system evolves to a stable state with a greater degree of cooperation. The results indicate that higher parameter *δ* can promote cooperation. Parameters are set to *β* = 2, *α* = 0.5 and *L* = 200.

We further investigated the impact of the parameter *δ* on strategic evolution from the perspective of population dynamics. Fig 2 shows the temporal evolution of cooperation for *b* = 1.1 under different *δ* values. The process exhibits two distinct stages: an initial decline followed by a rise. After reaching a minimum, the cooperation level increases steadily until it stabilizes at an evolutionary equilibrium. This characteristic dip-and-rise pattern reflects the intense competition between cooperators and defectors, which is typical of network reciprocity [41–44]. The proposed moderated strategy-update rule significantly enhances cooperation. Notably, higher *δ* values drive the system toward a more cooperative equilibrium, accelerate the evolution of cooperation, and shorten the duration of the second phase. These results confirm that the modified rule effectively promotes cooperation.

**Fig 3:**
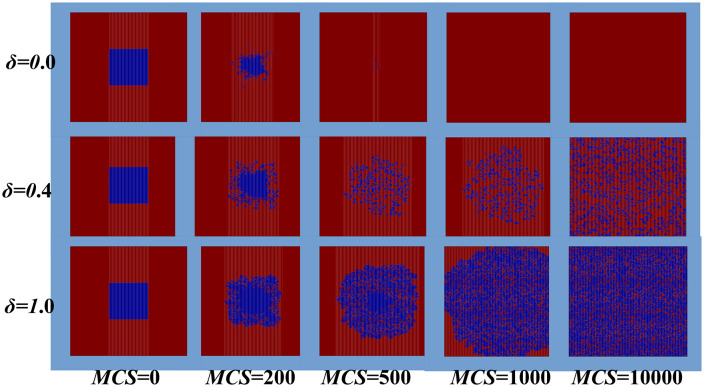
Shows snapshots of spatial distributions for blue cooperators and purple defectors at different *MCS* for various *δ* value. Vertically, the diffusion of cooperative behavior accelerates with increasing *δ*. Simulations indicate that larger *δ* significantly promotes cooperation. Parameters are set to *b* = 1.1, *α* = 0.5, *β* = 2 and *L* = 200.

Moving forward, we analyze the evolutionary dynamics by examining the spatial distribution of individual strategies to observe the competition between cooperative and competitive behaviors. As shown in Fig 3, when cooperators are initially placed in distinct positions, they quickly disappear under traditional strategy update rules. However, when moderated strategy update rules are applied, cooperators gradually spread within clusters of defectors. They gain a competitive advantage over defectors, which strengthens as parameter *δ* increases. Ultimately, this advantage allows cooperators to dominate, leading to a higher level of system cooperation at equilibrium. Notably, in the stable state, cooperators survive within numerous small, compact clusters.

Then, we further analyze the impact of sensitivity parameter *β* on cooperative evolution given parameter *δ =* 1*.0* and *α* = 0.5. And the impact of the system consistency aspiration or goal value *α* on cooperative evolution under the given parameter *β = 2* and *δ* = 1.0.

**Fig 4:**
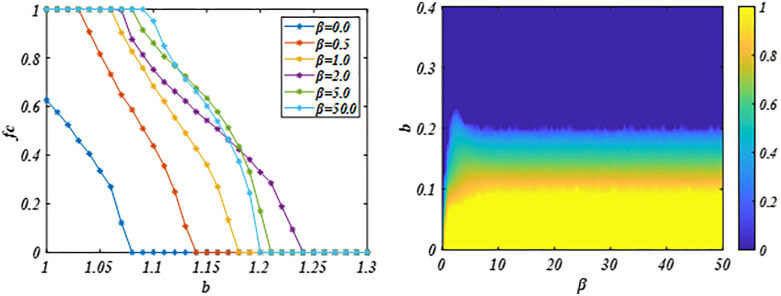
Panel (a) plots the *fc* against the *b* for different *β* values. Panel (b) demonstrates the dynamic impact of the *β* on cooperation evolution, revealing that while higher *β* values promote cooperation, the effect is only significant up to a certain threshold. The results indicate that a modestly sensitivity parameter *β* can promote cooperation. Parameters are set to *α* = 0.5, *δ* = 1.0 and *L* = 200.

In Fig 4(a), at *δ* = 1.0 and *α* = 0.5, the system cooperation level increases with the sensitivity parameter β, demonstrating its impact on cooperative evolution. This may be because the increase in sensitivity parameters amplifies the gap between game benefits and consistency aspiration or goals. Under this evaluation criterion, the results of cooperative payoff evaluation are amplified while the results of defective payoff evaluation are compressed, which accelerates the propagation and diffusion of cooperative behavior in the system, which also explains the strengthening effect of the proposed mechanism on cooperative behavior. However, as the sensitivity parameter *β* increases further, it suppresses cooperation in systems characterized by strong temptation. Under high-intensity social dilemma (*b* is large), defection obtain greater payoff that can achieving system goals. This overestimation of defection’s benefits facilitates its spread, thereby reducing overall cooperation. Fig 4(b) reveals the nonlinear dynamic influence of *β* on the evolution of cooperation. Thus, we can know that a modestly sensitivity parameter *β* can promote cooperation.

**Fig 5:**
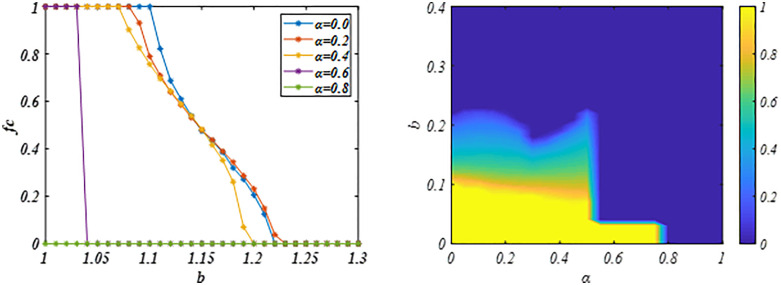
Panel (a) plots the *fc* against the *b* under different *α* values, Panel (b) shows the dynamic impact of *α* on the cooperative evolution, demonstrating that high consistency aspiration or goal suppress the propagation of cooperative behavior in the system. The results indicate that lower system consistency desires or goals can promote cooperation. Parameters are set to *β* = 2, *δ =* 1.0 and *L* = 200.

Fig 5(a) further examines the influence of the aspiration level *α* on the evolution of cooperation. The results show that lower system goals improve goal fulfillment among cooperators, thus promoting cooperation. However, an increase in the temptation *b* triggers an invasion of defectors, leading to a monotonic decline in system-wide cooperation. Despite this decrease, cooperation remains substantially higher than in traditional scenarios. Furthermore, cooperation diminishes as *α* rises. This is likely because higher *α* values make it more difficult for cooperative individuals to meet expectations. Under such evaluation criteria, the performance of individuals who fail to achieve *α* is accentuated, which hinders the propagation of cooperation. Therefore, as illustrated in Fig 5(b), setting lower system aspiration levels (or goals) can facilitate the spread of cooperation within this framework.

**Fig 6:**
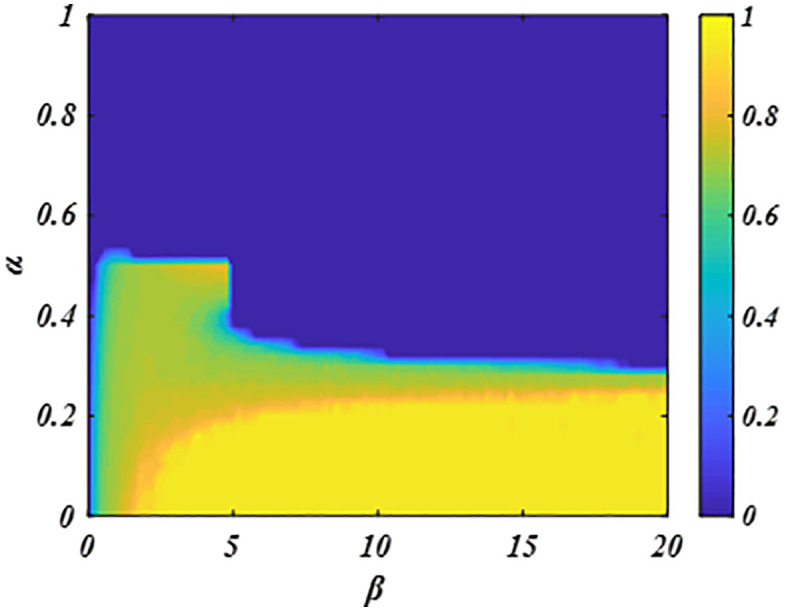
Illustrates the dynamic evolution of *fc* with parameter *β*-*α.* Horizontally, system cooperation increases as *β* increases, whereas vertically, it decreases as *α* increases. The results indicate that parameters *β* and *δ* jointly determine the cooperative evolution in the system. Parameters are set to *δ =* 1.0 and *L* = 200.

Further analysis in Fig 6 explores the dynamic influence of parameters *β* and *δ* on the evolution of cooperation. Horizontally, increasing the sensitivity parameter *β* alleviates the negative effect of aspiration level setting on cooperation within the system. Although this enhancement promotes collaborative behavior, its extent remains limited. Vertically, establishing appropriate aspiration levels is crucial for both initiating and maintaining cooperation. Thus, these parameters jointly shape the evolution of cooperation in the system.

**Fig 7:**
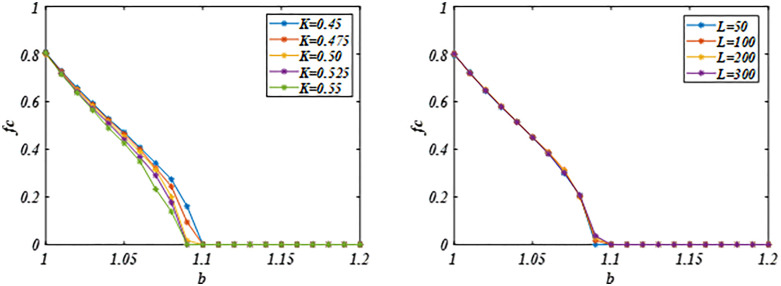
Panel (a) plots the *fc* against the *b* under different *K* values. Panel (b) plots the *fc* against the *b* under different *L* values. The result is consistent. The system exhibits robustness against minor noise interference and differences in network scale. Which indicating that the system is robust. Parameters are set to *β* = 2, *α* = 0.5, δ = 0.2 and *L* = 200 in Panel **(a)**, and *β* = 2, *α* = 0.5, *δ =* 0.2 and *K* = 0.5 in Panel (b).

To better illustrate the impact of parameter *K* on cooperative evolution, we calculated the evolutionary dynamics of the system for several values of *K*. As shown in Fig 7, the results indicate that the system maintains robustness under small noise fluctuations. However, when the noise amplitude becomes large, cooperative evolution is significantly disrupted. This finding is consistent with the conclusion reported by [40]. Moreover, analysis of the evolution of system cooperation across different network sizes (*L* = 50–300) shows consistent cooperation evolution, indicating that the system is robust.

## 4. Conclusion

Cooperation is recognised as foundation for sustaining socio-economic development. Building upon prior research and empirical observations of individual behavior within real social systems, this study introduces a novel strategy update rule. This rule incorporates a system-wide consensus objective and employs an individual’s historical attainment of this objective as a key performance metric, calculated using the Bush-Mosteller (BM) model within a reinforcement learning framework.

Results demonstrate that, compared to conventional update mechanisms, the proposed rule significantly amplifies cooperative behavior within the system. This enhancement stems from the rule’s systematic devaluation of defection payoffs while concurrently amplifying the perceived benefits of cooperation. This direct mechanism effectively suppresses the proliferation of defection strategies and accelerates the diffusion of cooperative ones. Furthermore, the inherent multidimensionality of the composite performance metric constrains opportunities for defectors by selectively filtering potential imitators. This aligns with empirical evidence indicating that social evaluations rarely hinge on a singular criterion. Critically, establishing an appropriate evaluative benchmark directly fosters cooperative outcomes within groups, and the methodology for behavioral assessment relative to this benchmark is paramount for optimizing group management. Collectively, our findings suggest that multidimensional evaluation creates more favorable conditions for the emergence and persistence of cooperation within complex environmental systems. This research offers theoretical insights into the mechanisms underpinning cooperative behavior in collective settings.

## Supporting information

S1 File This code is used to calculate Fig 1. (TXT)

S2 File This code is used to calculate Fig 2. (TXT)

S3 File This code is used to calculate Fig 3. (TXT)

S4 File This code is used to calculate Fig 4(a). (TXT)

S5 File This code is used to calculate Fig 4(b). (TXT)

S6 File This code is used to calculate Fig 5(a). (TXT)

S7 File This code is used to calculate Fig 5(b). (TXT)

S8 File This code is used to calculate Fig 6. (TXT)

S9 File This code is used to calculate Fig 7(a). (TXT)

S10 File This code is used to calculate Fig 7(b). (TXT)

S11 File Data of all figure. (ZIP)
